# Comparative study on the immunogenicity between an HLA-A24-restricted cytotoxic T-cell epitope derived from survivin and that from its splice variant survivin-2B in oral cancer patients

**DOI:** 10.1186/1479-5876-7-1

**Published:** 2009-01-06

**Authors:** Jun-ichi Kobayashi, Toshihiko Torigoe, Yoshihiko Hirohashi, Satomi Idenoue, Akihiro Miyazaki, Akira Yamaguchi, Hiroyoshi Hiratsuka, Noriyuki Sato

**Affiliations:** 1 Department of Pathology, Sapporo Medical University School of Medicine, Sapporo, Japan; 2 Department of Oral Surgery, Sapporo Medical University School of Medicine, Sapporo, Japan; 3 Department of Surgery, Sapporo Medical University School of Medicine, Sapporo, Japan

## Abstract

**Background:**

We previously reported an HLA-A24-restricted cytotoxic T-cell epitope, Survivin-2B80-88, derived from a splice variant of survivin, survivin-2B. In this report, we show a novel HLA-A24-restricted T-cell epitope, Survivin-C58, derived from a wild type survivin, and compared their immunogenicity in oral cancer patients.

**Methods:**

By stimulating peripheral blood lymphocytes of HLA-A24-positive cancer patients with Survivin-C58 peptide *in vitro*, the peptide-specific CTLs were induced. In order to compare the immunogenic potential between C58 peptide and 2B80-88 peptide, peripheral blood T-cells from thirteen HLA-A24-positive oral cancer patients were stimulated with either or both of these two peptides.

**Results:**

Survivin-2B80-88 peptide-specific CTLs were induced from four patients, and C58 peptide-specific CTLs were induced from three out of eight patients with over stage II progression. The CTLs exerted cytotoxicity against HLA-A24-positive tumor cells. In contrast, CTL induction failed from a healthy volunteer and all four patients with cancer stage I.

**Conclusion:**

It was indicated that a splicing variant-derived peptide and wild type survivin-derived peptide might have a comparable potency of CTL induction, and survivin targeting immunotherapy using survivin-2B80-88 and C58 peptide cocktail should be suitable for HLA-A24+ oral cancer patients.

## Background

Survivin, an inhibitor of apoptosis protein, is highly expressed in the vast majority of cancers [1,2]. Survivin has been shown to increase tumor resistance to apoptotic stimuli, such as radiation and chemotherapy [3,4]. In agreement with these findings, a number of reports demonstrate that survivin expression in cancer cells has a prognostic value and is associated with increased tumor recurrence and shorter patient survival [5-10], although the opposite correlation is reported in certain cancers [11]. So far, four different splicing variants of human survivin have been described, including survivin-2α, survivin-2B, survivin-ΔEx3, and survivin-3B [12-15]. While survivin-2α and survivin-3B are truncated forms, survivin-2B results from alternating splicing at the interface between exon 2 and exon 3, leading to insertion of an additional exon, termed exon 2B, in BIR domain. Since BIR domain is a functional domain that is important for the anti-apoptotic activity of survivin, survivin-2B is predicted to be non-anti-apoptotic [16,17].

Survivin was originally detected only in normal thymus, testis and placenta; however, low levels of wild type survivin was detected in other normal tissues, such as activated T-cells, vascular endothelial cells, and hematopoietic cells by more sensitive methods [3,18,19]. Wild type survivin is known to have an essential role in the mitosis [3,18,20]. It forms a complex with the chromosomal passenger proteins during mitosis and regulates mitotic progression. In contrast, the protein levels as well as the mRNA levels of survivin-2B and other survivin variants are far less than that of wild type survivin, and they are dispensable in such a mitotic checkpoint [17,21].

Since survivin expression is very low in normal differentiated adult tissues as compared with that in cancer tissues, survivin is considered to be an ideal molecular target for cancer immunotherapy. With this mind, we attempted to identify a HLA-A24-restricted cytotoxic T-lymphocyte (CTL) epitopes of survivin that were suitable for cancer vaccine, since HLA-A24 was the most frequent allele in Japanese. In our previous report, three peptides derived from survivin and its splicing variant survivin-2B were examined for HLA-A24-binding affinity and immunogenicity [22]. It was shown that Survivin-2B80-88 peptide (amino acid sequence AYACNTSTL), which was derived from a splicing variant survivin-2B-specific exon2B, was capable of inducing CTLs that had killing activity to HLA-A24^+ ^cancer cells. Following this report, we provided further evidence that Survivin-2B80-88 was highly immunogenic in various cancer patients, including those with gastric cancer, breast cancer, and colorectal cancer [23]. Based on these results *ex vivo*, we have conducted phase I clinical trials assessing the adverse event and efficacy of Survivin-2B80-88 peptide vaccination in patients with advanced colorectal cancer, breast cancer, lung cancer, bladder cancer, and oral cancer [24-26].

Though we failed to identify an HLA-A24-restricted CTL epitope derived from wild type survivin in the initial study, a number of epitopes have been identified from wild type survivin that are restricted to other HLA class I alleles, such as A1, A2, A11, and B35 [27-29], some of which have been applied for clinical trials [30,31]. More recently, Andersen, et al. demonstrated that wild type survivin-derived Sur20-28 peptide (amino acid sequence STFKNWPFL) was capable of inducing the peptide-specific CD8-positive T-cells from PBMCs of HLA-A24^+ ^cancer patients, although HLA-A24-restricted killing activity of the peptide-specific T-cells against survivin-positive cancer cells has not been assessed [32]. In this study, we present a novel CTL epitope Survivin-C58 peptide derived from wild type survivin. The peptide-specific CTLs induced from peripheral blood mononuclear cells (PBMCs) of oral cancer patient exerted HLA-A24-restricted cytotoxicity against the tumor cells. Then, we stimulated PBMCs of oral cancer patients with either or both Survivin-C58 and Survivin-2B80-88 peptides, and the consequent CTLs were examined for the peptide-specificity and cytotoxicity against HLA-A24+ tumor cells. We demonstrate here for the first time a comparative study on the potency of inducing CTLs *in vitro *between wild type survivin-derived peptide and survivin-2B-derived peptide, which indicates the comparable potency of CTL induction in oral cancer patients.

## Materials and methods

### Patients and samples

Surgically-resected cancer specimens and PBMCs used in this study were obtained from HLA-A*2402^+ ^patients with breast cancer or oral cancer who were hospitalized at Sapporo Medical University Hospital after obtaining their informed consent. The patients' clinicopathological profiles were listed on the table.

### Cell lines and culture media

Human breast cancer cell lines, HMC-1 and HMC-2, human oral squamous cell carcinoma (OSCC) cell lines, OSC19, OSC20, OSC30, OSC40, OSC70, and POT1 were established in our laboratory. OSCC cell lines HO-1-NH, KOSC-3, HSC-2, HSC- 3, and HSC-4 were purchased from the Human Science Research Resources Bank (HSRRB, Osaka, Japan). OSCC cell line SAS was obtained from the Institute of Development, Aging and Cancer Tohoku University (Tohoku, Japan). Human embryonic kidney cell line 293T, breast cancer cell line MCF7, lymphoma cell line Daudi and leukemia cell line K562 were purchased from American Type Culture Collection (Manassas, VA). C1R-A24 and C1R-A31, lymphoblastoid cell line C1R transfectants with HLA-A*2402 and HLA-A*31012 cDNA respectively, were kind gifts from Dr. M. Takiguchi (Kumamoto University School of Medicine, Kumamoto, Japan). T2-A24, a stable transfectant of T2 cells with HLA-A*2402 cDNA was a kind gift from Dr. K. Kuzushima (Aichi Cancer Research Institute, Nagoya, Japan).

293T cells and breast cancer lines were cultured in Dulbecco's modified Eagle's medium (DMEM, Sigma-Aldrich, St. Louis, MO) with 2 mM L-glutamine, 10% heat-inactivated fetal bovine serum, 100 U/ml penicillin G, and 100 mg/ml streptomycin at 37°C in humidified 5% CO_2 _atmosphere. All the OSCC cell lines were cultured in RPMI 1640 (Sigma-Aldrich, St. Louis, MO) medium supplemented with 2 mM L-glutamine, 10% fetal bovine serum, 100 U/ml penicillin G, and 100 μg/ml streptomycin at 37°C in a humidified 5% CO2 atmosphere. OSC20-A24, a stable transfectant of OSC20 with HLA-A*2402 cDNA was cultured in a medium supplemented with 800 ng/ml of puromycin (Sigma-Aldrich, St. Louis, MO). Hygromycin B (0.5 mg/ml, WAKO chemicals, Osaka, Japan) or G418 (800 μg/ml, GIBCO/Invitrogen Corp., Carlsbad, CA) was continuously added to the culture medium for C1R transfectants and T2 transfectant, respectively.

### RT-PCR Analysis

A set of total RNA from normal human adult tissues was purchased from Clontech (human total RNA master panel). Total RNA was isolated from cultured cells by using ISOGEN reagent (Nippon Gene, Tokyo, Japan). The cDNA mixture was synthesized from 1 mg of total RNA by reverse transcription using Superscript II and oligo(dT) primer (Life Technologies, Inc., Gaithersburg, MD) according to the manufacturer's protocol. PCR amplification was performed in 50 ml of PCR mixture containing 1 mL of the cDNA mixture, KOD Plus DNA polymerase (Toyobo, Osaka, Japan), and 50 pmol of primers. The PCR mixture was initially incubated at 94°C for 2 min, followed by 30 cycles of denaturation at 94°C for 15 s, annealing at 57°C for 30 s, and extension at 68°C for 1 min. Primer pairs used for RT-PCR analysis were 5'-TCAAGGACCACCGCATCTCTAC-3' and 5'-GCACTTTCTTCGCAGTTTCCTC-3' as a forward and a reverse primer, respectively. Expected sizes of PCR products for wild type survivin, survivin-2B, and survivin-DEx3 were 355 bp, 424 bp, and 236 bp, respectively. As an internal control glyceraldehyde-3-phosphate dehydrogenase (G3PDH) was detected by using a forward primer 5'-ACCACAGTCCATGCCATCAC-3' and a reverse primer 5'-TCCACCACCCTGTTGCTGTA-3' with an expected PCR product of 452 bp. The PCR products were visualized with ethidium bromide staining under UV light after electrophoresis on 1.0% agarose gel. Nucleotide sequence of the PCR products was confirmed by direct sequencing using ABI Genetic analyzer PRIM 310 and an AmpliCycle sequencing kit (Perkin-Elmer, Foster City, CA).

### Western blotting

Cultured cells were washed in ice-cold PBS, lysed by incubation on ice in a lysis buffer [50 mmol/L Tris-HCl (pH 8.0), 150 mmol/L NaCl, 1% NP40, protease inhibitor cocktail; Complete, Roche Diagnostics, Inc., Basel, Switzerland], and clarified by centrifugation at 15,000 rpm for 20 minutes at 4°C. The whole-cell lysates were boiled for 5 minutes in the presence of SDS sample buffer, resolved by 10% SDS-PAGE, and electrophoretically transferred to polyvinylidene difluoride (PVDF) membranes (Immobilon-P, Millipore, Billerica, MA). The membranes were then incubated with blocking buffer (5% nonfat dry milk in PBS) for 1 hour at room temperature and then incubated for 40 minutes with mouse anti-human survivin monoclonal antibody (Santa Cruz Biotechnology) or mouse anti-β-actin monoclonal antibody AC-15 (Sigma-Aldrich). After washing three times with wash buffer (0.1% Tween-20 in PBS), the membrane was reacted with peroxidase-labeled goat anti-mouse IgG antibody (KPL, Gaithersburg, MD) for two hours. Finally, the signal was visualized by using an enhanced chemiluminescence (ECL) detection system (Amersham Life Science, Arlington Heights, IL) according to the manufacturer's protocol.

### Peptides and Cytokines

Wild type survivin-derived peptides carrying HLA-A24 binding motif Survivin-C58 (amino acid sequence FFCFKELEGW), a splicing variant survivin-2B-derived peptide Survivin-2B80-88 (AYACNTSTL) [22], EBV LMP2-derived HLA-A24 binding peptide (TYGPVFMSL) [33], HIV env-derived HLA-A24 binding peptide (RYLRDQQLLGI) [34], CMV pp65-derived HLA-A24 binding peptide (QVDPVAALF), mouse VSV-derived peptide VSV8 (RGYVYQGL), and synovial sarcoma chromosomal translocation product SYT-SSX-derived SS393 peptide and K9I peptide (GYDQIMPKK and GYDQIMPKI respectively) [35,36] were purchased from SIGMA Genosys (Ishikari, Japan). They were resolved in DMSO at the concentration of 5 mg/ml and stored at -80°C. Human recombinant interleukin (IL)-2 was a kind gift from Takeda Pharmaceutical Co. (Osaka, Japan). Human recombinant GM-CSF was a kind gift from Kirin (Tokyo, Japan) and Novartis Pharmaceutical (Basel, Switzerland). Human recombinant IL-4 and IL-7 were purchased from Invitrogen (San Diego, CA).

### Peptide Binding Assay

Peptide binding affinity to HLA-A24 was assessed by HLA-A24 stabilization assay as described previously [22]. Briefly, after incubation of T2-A24 cells in culture medium at 26°C for 18 h, cells (2 × 10^5^) were washed with PBS and suspended with 1 ml of Opti-MEM (Life Technologies, Inc.) containing 3 μg/ml of β 2-microglobulin with or without 100 μg of peptide, followed by incubation at 26°C for 3 h and then at 37°C for 3 h. After washing with PBS, the cells were incubated with anti-HLA-A24 monoclonal antibody (c7709A2.6, kindly provided by Dr. P. G. Coulie, Ludwig Institute for Cancer Research, Brussels Branch) at 4°C for 30 min, followed by incubation with FITC-conjugated rabbit anti-mouse IgG at 4°C for 30 min. The cells were then suspended with 1 ml of PBS containing 1% formaldehyde and analyzed by FACScan (Becton Dickinson, Mountain View, CA). Binding affinity was evaluated by comparing mean fluorescence intensity of HLA-A24 expression in the presence of peptide pulsation to mean fluorescence intensity in the absence of the peptide.

### Peptide specific CTL induction with immature dendritic cells and phytohemagglutinin blasts

CTLs were induced from PBMCs by using autologous dendritic cells (DCs) and phytohemagglutinin (PHA) blasts as antigen presenting cells (APC). Briefly, PBMCs were isolated from one healthy volunteer and 12 cancer patients (one breast cancer and eleven oral cancer) by standard density gradient centrifugation on Lymphoprep (Nycomed, Oslo, Norway) and cultured in AIM-V medium (Life Technologies) at 37°C for 2 h to separate adherent cells and non-adherent cells. Autologous immature DCs were generated from adherent cells in the plastic flask by culturing in AIM-V medium supplemented with HEPES (10 mmol/L), 2-mercaptoethanol (50 μmol/L), GM-CSF (1000 units/mL) and IL-4 (1000 units/mL) for 7 days. CD8^+ ^cells were isolated from non-adherent cells in the plastic flask by the MACS separation system (Miltenyi Boitech, Bergish Blabach, Germany) using anti-CD8 monoclonal antibody coupled with magnetic microbeads according to manufacturer's instruction. PHA blasts were derived from CD8^- ^cells by culturing in AIM-V medium containing IL-2 (100 units/mL) and PHA (1 μg/mL, Wako Chemicals, Osaka, Japan) for 3 days, followed by washing and cultivation in the presence of IL-2 (100 units/ml) for 4 days. DCs and PHA blasts were cultured in AIM-V medium supplemented with 50 μmol/L of peptide at room temperature for 2 h, washed with AIM-V, and then irradiated (100 Gy) before use.

CTL induction procedure was initiated by stimulating 2 × 10^6 ^CD8^+ ^cells with peptide-pulsed autologous DCs at a 20:1 effecter/APC ratio in AIM-V supplemented with IL-7 (10 ng/mL) for 7 days at 37°C. The following stimulation was performed with peptide-pulsed PHA blasts at a 5:1 effector/APC ratio. On the next day of the second stimulation, IL-2 was added to the culture at a concentration of 50 units/mL. The same CTL stimulation cycle with PHA blasts was then performed twice more over the period of two weeks. One week after the 4th stimulation, cytotoxic activity of the CTLs was measured by ^51^Cr release assay.

### Cytotoxicity assay

The cytotoxic activities of CTLs were measured by ^51^Cr release assay as described previously [22]. Briefly, target cells were labeled with 100 μCi of ^51^Cr for 1 hr at 37°C, washed thrice, and resuspended in AIM-V medium. Then, 3 × 10^3 ^^51^Cr-labeled target cells were incubated with effecter cells at various effector/target (E/T) ratios at 37°C for 6 h in V-bottom 96-well microtiter plates. Then supernatants were collected and the radioactivity was measured by a gamma-counter. The percentage of specific lysis was calculated as following: % specific lysis = (test sample release - spontaneous release) × 100/(maximum release - spontaneous release). For preparation of peptide-pulsed target cells, T2-A24 cells or C1R-A24 cells were incubated with 50 μg/mL of peptide at room temperature for 2 h before the assay. For preparation of tumor target cells, target cells were treated with 100 units/ml of IFN-γ for 48–72 h before the assay.

## Results

### Survivin expression in oral cancer cells

**Figure 1:**
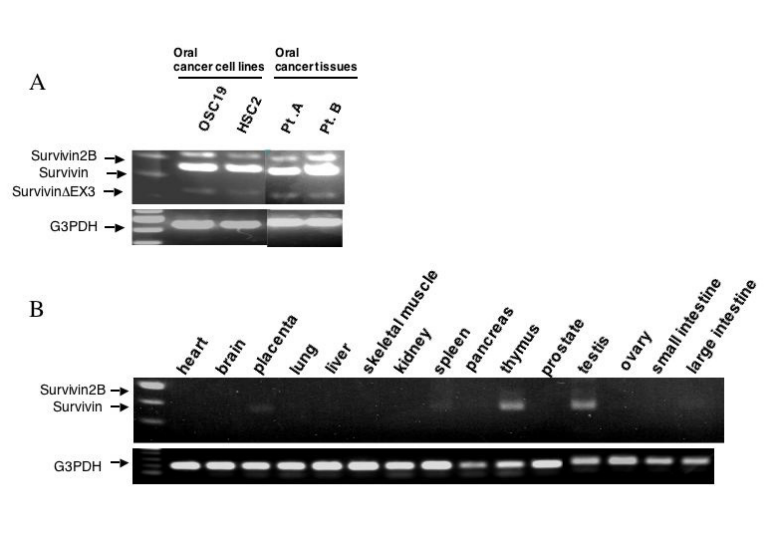
**Expression of survivin mRNA as assessed by RT-PCR in normal tissues, and oral cancer cell lines and primary oral cancer tissues**. **(A) **Expression of survivin mRNA in oral cancer cell lines and primary oral cancer tissues from two patients. G3PDH expression was detected as an internal control. **(B) **Expression of survivin mRNA in normal adult tissues. 293T cells transfected with myc-tagged survivin cDNA (293T-survivin) was used as a positive control for survivin expression. G3PDH expression was detected as an internal control.

We previously showed that the survivin mRNA level was elevated in various cancer cell lines, including gastric cancer cells, colon cancer cells, breast cancer cells, lung cancer cells, bladder cancer cells, renal cancer cells, and melanoma cells [22]. In the present study, we focused on the survivin expression in oral cancer cell lines. In concurrence with previous reports [5], survivin was highly expressed in oral cancer tissues as well as oral cancer cell lines. In the RT-PCR analysis, three bands were detected, corresponding to survivin-2B, wild type survivin, and survivin-ΔEx3 respectively (Fig. 1A), which were confirmed by DNA sequence analysis. By the same RT-PCR method, wild type survivin expression was detected only in the placenta, thymus, and testis among normal adult tissues; however, survivin-2B and survivin-ΔEx3 were barely detected (Fig. 1B). By using more sensitive RT-PCR analysis, expression of these splicing variants was shown only in the thymus [22].

**Figure 2:**
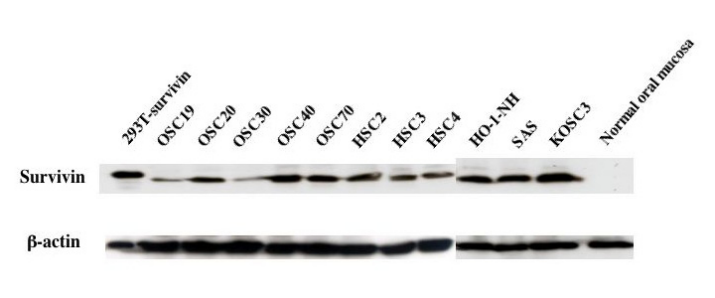
**Western blotting analysis of survivin protein in oral cancer cell lines**. Lysates from oral cancer cell lines or normal oral mucosal tissue were resolved by 10% SDS-PAGE and transferred to PVDF membranes. The membranes were then incubated with mouse anti-human survivin monoclonal antibody (upper panel) or mouse anti-β-actin monoclonal antibody AC-15 (lower panel).

We then analyzed the survivin expression in the protein level. In all the oral cancer cell lines examined in the present study, wild type survivin was detected, but not in normal oral mucosal tissue by Western blotting (Fig. 2). A small amount of survivin-2B protein was also detected in some cell lines. These data indicate that the expression of survivin-2B was more restricted to cancer tissues, though its level was far less as compared to that of wild type survivin.

### HLA-A24-binding analysis of survivin-derived peptides

**Figure 3:**
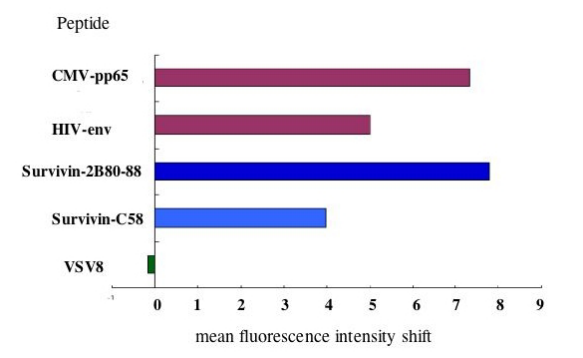
**HLA-A24-binding assay of peptides**. Binding affinity of peptide to HLA-A24 molecule was evaluated by mean fluorescent intensity (MFI) shift of cell surface HLA-A24 level on T2-A24 cells that were pulsed with each peptide. CMV pp65-derived HLA-A24-binding peptide (QVDPVAALF) and HIV env-derived HLA-A24-binding peptide (RYLRDQQLLGI) were used as positive controls. VSV-derived peptide VSV8 (RGYVYQGL) was used as a negative control. Histograms of MFI shift were displayed for each peptide. MFI shift was calculated as; MFI shift = (MFI of T2-A24 cells pulsed with the peptide) - (MFI of T2-A24 cells without peptide pulsation).

To evaluate if wild type survivin might become a target of immunotherapy as well as a splicing variant survivin-2B, we re-screened the total amino acid sequence of wild type survivin protein for peptides containing HLA-A24-binding motif. In our previous report, two peptides, survivin85-93 and survivin92-101, derived from exon 3-encoded region were examined; however, they did not have a significant binding affinity to HLA-A24 [22]. In the present study, we identified another peptide, designated as survivin-C58 (amino acid sequence FFCFKELEGW), which was derived from exon 2-encoded region. Survivin-C58 and survivin-2B80-88 were assessed for the binding ability to HLA-A24 molecule by HLA stabilization assay using transporters associated with antigen processing (TAP) deficient and HLA-A*2402-transfected cell line, T2-A24 cells, as described previously [35,36]. Two positive control peptides, HLA-A24-restricted CMV-pp65 epitope and HIV-env epitope, and a negative control peptide VSV8 were used in the assay. HLA-A24 level on the cell surface of T2-A24 cells is up-regulated in the presence of HLA-A24-binding peptides. Up-regulation of mean fluorescence intensity (MFI) of cell surface HLA-A24 was detected by flow cytometer (Fig. 3). Both CMV-pp65-derived peptide and HIV-env-derived peptide increased MFI of HLA-A24 clearly, while VSV8-derived peptide failed, indicating adequate qualification of this assay. Both survivin-2B80-88 and survivin-C58 peptides were capable of up-regulating the HLA-A24 levels, though survivin-C58 showed less binding capacity than survivin-2B80-88.

### CTL induction from PBMCs of HLA-A*2402^+ ^cancer patients

In order to know if HLA-A24-restricted peptide-specific CTLs are induced from PBMCs of cancer patients, PBMCs were collected from HLA-A*2402-positive cancer patients (one breast cancer patient and one oral cancer patient), and stimulated *in vitro *with survivin-C58 peptide in the presence of autologous monocyte-derived DC or autologous PHA blasts. After 4 times stimulation, cytotoxic activity against peptide-pulsed target cells was examined by ^51^Cr release assay. As shown in Fig. 4A, CTLs induced from PBMCs of a breast cancer patient were capable of killing survivin-C58-pulsed T2-A24 target cells, but they failed in killing SYT-SSX-derived peptide-pulsed T2-A24 cells or survivin-C58-pulsed HLA-A24-negative target cells. The same CTLs showed a significant cytotoxicity to HLA-A*2402-positive breast cancer cells, HMC2 and HMC1, but not to HLA-A*2402-negative breast cancer cells MCF7. The similar result was shown in Fig. 4B, when CTLs were induced from an oral cancer patient (Case #13 in Table 1). Survivin-C58 peptide-specific CTLs showed cytotoxicity against HLA-A*2402-transfected oral cancer cell line OSC20. Therefore, it was indicated that wild type survivin-derived survivin-C58 peptide could be presented on tumor cells in the context of HLA-A24 and recognized by CTLs.

**Table 1:** CTL induction from PBMCs of oral cancer patients

						CTL induction		Survivin
Case no.	age	sex	stage	Origin, histology	Prior treatment	2B80-88 specific CTL	Survivin-C58-specific CTL	expression

#1	63	M	Stage II	buccal mucosa, SCC, well	Chem, Surg	-	+	+
#2	69	M	Stage I	tongue, SCC, basaloid	Surg	-	-	+
#3	60	F	Stage II	mandibular, SCC, m	Chem, Surg	-	-	+
#4	60	M	Stage III	oropharynx, SCC, mod	Chem, Surg	+	-	+
#5	50	F	Stage II	tongue, SCC, well	Chem, Surg	+	+	+
#6	83	F	Stage IVA	maxillary gingiva, SCC, well	Chem, Surg	-	-	+
#7	64	M	Stage II	tongue, SCC, well	Chem, Surg	+	n.d.	+
#8	50	F	Stage II	submaxillary gland, Adenoid cystic	Chem, Surg	-	-	+
#9	65	F	Stage I	tongue, SCC, well	Surg	-	-	+
#10	66	F	Stage II	tongue, SCC, mod	Chem, Surg	+	-	+
#11	73	F	Stage I	tongue, SCC, well	Surg	-	-	+
#12	50	F	Stage I	tongue, SCC, well	Surg	-	-	+
#13	67	M	Stage IVA	oral floor, SCC, poorly	Chem, Surg	n.d.	+	+
**H#1**	35	M	-	healthy volunteer	-	-	-	

**Figure 4:**
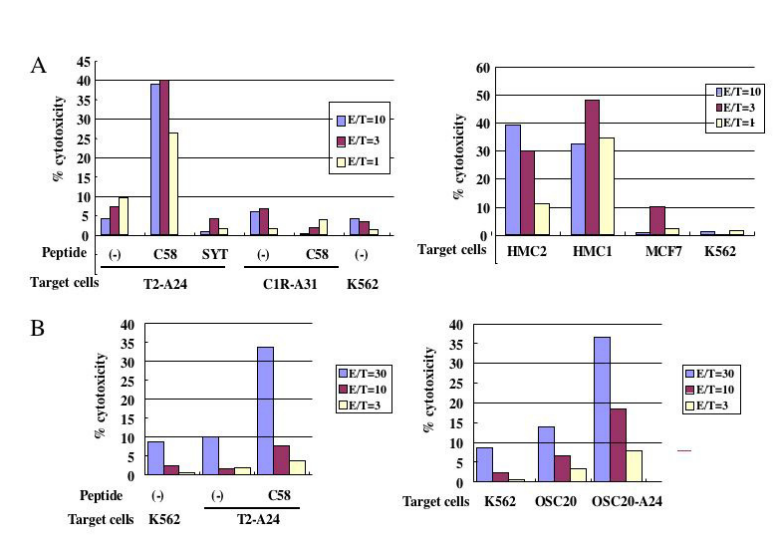
**Induction of survivin-C58 peptide-specific CTLs and their cytotoxicity against survivin-positive cancer cell lines**. CTLs were induced from PBMCs of an HLA-A*2402^+ ^breast cancer patient by stimulating with survivin-C58 peptide-pulsed APCs. After four times stimulation, CTLs were subjected to standard ^51^Cr release assay at the indicated effector/target (E/T) ratio. In the left panel, T2-A24 cells and C1R-A31 cells were pulsed with or without survivin-C58 peptide (C58) or SYT-SSX-derived SS393 peptide (SYT), serving as target cells. In the right panel, survivin-positive breast cancer cell lines with HLA-A*2402 (HMC1 and HMC2) or without HLA-A*2402 (MCF7 and K562) were used as target cells. **(A) **CTLs were induced from PBMCs of an HLA-A*2402^+ ^oral cancer patient (case #13 in Table 1) by stimulating with survivin-C58 peptide-pulsed APCs. After four times stimulation, CTLs were subjected to standard ^51^Cr release assay at the indicated effector/target (E/T) ratio. In the left panel, T2-A24 cells were pulsed with or without survivin-C58 peptide (C58), serving as target cells. In the right panel, survivin-positive HLA-A*2402-negative oral cancer cells (OSC20) and OSC20 transfectants with HLA-A*2402 cDNA (OSC20-A24) were used as target cells.

### CTL induction efficiency with survivin-2B80-88 or survivin-C58 from PBMCs of HLA-A24+ oral cancer patients

Previously we showed that survivin-2B80-88-specific CTLs were induced efficiently from PBMCs of HLA-A24+ patients with survivin-positive breast cancer, colorectal cancer, and gastric cancer [23]. In the present study, we examined if survivin-2B80-88-specific CTLs and survivin-C58-specific CTLs could be induced from PBMCs of HLA-A24+ oral cancer patients. PBMCs were collected from thirteen patients with survivin-positive oral cancer and one healthy volunteer with HLA-A*2402 genotype (Table 1), and stimulated with either or both of these two peptides *in vitro *in the presence of autologous DC or PHA blasts as APCs. After 4 times stimulation over a period of four weeks, CTLs were examined for their peptide-specific killing activity by 51-Cr release assay using peptide-pulsed T2-A24 target cells. Survivin-2B-specific CTLs were induced from four patients out of twelve patients examined, and survivin-C58-specific CTLs were induced from three patients out of twelve patients examined. Though the number of patients in this study was too few to discuss the exact correlation, it is possible that the CTL induction efficiency might be related to the disease progression stage of the patients, since CTLs could not be induced from any of four patients with stage I (cases #2, #9, #11, and #12), nor from a healthy volunteer.

**Figure 5:**
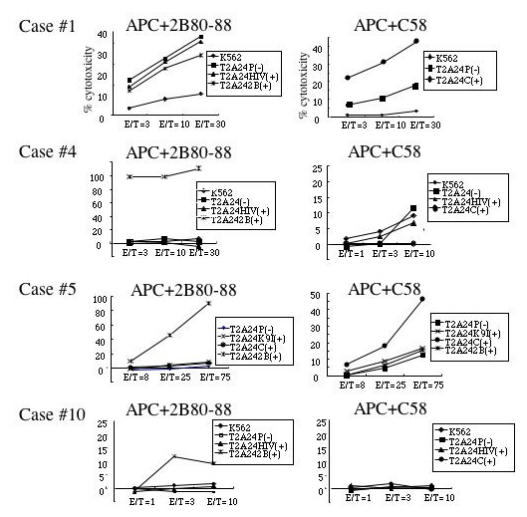
Peptide-specific CTL induction using survivin-2B80-88 peptide and survivin-C58 peptide from PBMCs of HLA-A*2402^+ ^oral cancer patients. PBMCs of HLA-A*2402^+ ^oral cancer patients were stimulated *in vitro *with survivin-2B80-88 peptide-pulsed APCs (APC+2B80-88) and survivin-C58 peptide-pulsed APCs (APC+C58) separately, followed by assessment of the peptide-specific cytotoxic activity by ^51^Cr release assay at the indicated effector/target (E/T) ratio. T2-A24 cells were pulsed with HIV-env peptide (HIV+), SYT-SSX-derived peptide (K9I+), survivin-2B80-88 peptide (2B+), or survivin-C58 peptide (C+), serving as target cells. P(-) indicates T2-A24 target cells without peptide pulsation. K562 target cells were used for monitoring natural killer activity and lymphokine-activated non-specific cytotoxicity.

PBMCs from eleven patients were stimulated with survivin-2B80-88 and survivin-C58 peptides in separate wells. CTLs with specificity to either of the two peptides were induced from three cases (case #1 specific to survivin-C58, and cases #4 and #10 specific to survivin-2B80-88), and both survivin-2B80-88-specific CTLs and survivin-C58-specific CTLs were successfully induced from one case (case #5) (Fig. 5). These data indicate that survivin-2B80-88 and survivin-C58 peptides have a comparable potency of CTL induction in oral cancer patients.

## Discussion

Survivin is overexpressed in a variety of cancer tissues, and at least four different splicing variants have been identified so far. Wild type survivin is known to have an important role in the mitotic checkpoint in normal cells and an anti-apoptotic function in cancer cells [3,18]. In contrast, the splicing variants are dispensable in the mitotic checkpoint [21], and anti-apoptotic function is lost in some splicing variants such as survivin-2B, in which BIR domain is disrupted by the insertion of exon 2B [17]. Survivin-2B and other splicing variant proteins are unstable in cells, thereby degraded rapidly. Therefore, survivin splicing variants do not appear to be suitable for the target molecules in targeting cancer therapy. However, survivin-2B is an attractive target antigen for cancer immunotherapy, since it contains a unique amino acid sequence and is barely expressed in normal adult tissue including thymus, where T-cell tolerance is induced. We have identified HLA-A24-restricted CTL epitope survivin-2B80-88 derived from survivin-2B previously and reported that it had a high potency of CTL induction in various cancer patients including breast cancer, colorectal cancer, and gastric cancer patients [23]. On the basis of these findings *in vitro*, clinical trials of survivin-2B80-88 peptide immunotherapy have been conducted for advanced cancers such as colorectal cancer, breast cancer, lung cancer, and oral cancer [24,26], in which tumor regression (partial response) was observed in certain cases. Other groups have identified the other HLA-restricted CTL epitopes from wild type survivin and applied for clinical trials [30,31]. More recently, a novel HLA-A24-restricted CTL epitope Sur20-28 was identified from wild type survivin by the screening of a peptide library of overlapping nonamers spanning the full length of survivin protein [32]. Though the peptide was shown to induce peptide-specific perforin-positive CD8+ T-cells from PBMCs of cancer patients, it remains to be determined whether the peptide-specific T-cells have a capability of killing cancer cells in an HLA-A24-restricted manner. However, it may be true that wild type survivin is also immunogenic to cancer host as well as its splicing variant survivin-2B. Therefore, we re-screened to find a novel CTL epitope derived from wild type survivin in the present study. Survivin-C58 peptide-specific CTLs were successfully induced from PBMCs of advanced oral cancer patients and exerted HLA-A24-restricted cytotoxicity against oral cancer cells. The CTL induction efficiency of survivin-C58 peptide was almost comparable to that of survivin-2B80-88 peptide, and it was noted that CTL could not be induced from PBMCs of oral cancer patients with stage I. These findings contrast with our previous report that survivin-specific CTLs were induced successfully from PBMCs of breast cancer patients and colorectal cancer patients with stage I [23]. It is speculated that immunogenicity of tumor-expressed survivin may be lower in the early oral cancer than that in other cancers. It is possible that the peptide-specific CTL efficiency might be related to the expression levels of survivin or survivin-2B proteins in the tumor tissues. As shown in Table 1, survivin expression was detected in all the cases by immunostaining. Though there were some differences in the staining intensity among the cases, we couldn't find any correlation between the staining intensity and the CTL induction efficiency.

Why does survivin have so immunogenic feature despite the abundant expression in thymus? The exact answer remains unknown. Interestingly, we observed that survivin-positive cells in thymus are mainly cortical thymocytes, but not medullary epithelial cells or dendritic cells that mediate negative selection and T-cell tolerance. It may explain at least in part the incomplete peripheral tolerance and immunogenic feature of survivin.

## Conclusion

In conclusion, we provided evidence that wild type survivin is an attractive target for the immunotherapy against oral cancer as well as survivin-2B, and survivin targeting immunotherapy using survivin-2B80-88 and C58 peptide cocktail should be suitable for HLA-A24+ cancer patients.

## Abbreviations

CTL: cytotoxic T-lymphocyte; PBMC: peripheral blood mononuclear cells; OSCC: oral squamous cell carcinoma; DC: dendritic cell; PHA: phytohemagglutinin; APC: antigen presenting cell.

## Competing interests

The authors declare that they have no competing interests.

## Authors' contributions

JK carried out the CTL induction, killing assays and drafted the manuscript. TT and YH participated in the design of the study and performed the evaluation of the data. TT helped to draft the manuscript. SI contributed to the HLA-A24-binding assay and CTL induction from PBMCs. AM, AY and HH contributed to collecting patients' samples with the informed consent. HH and NS contributed to the design and coordination of this study as well as reviewing the manuscript. All authors have read and approved the final manuscript.
